# Advanced extracellular vesicle bioinformatic nanomaterials: from enrichment, decoding to clinical diagnostics

**DOI:** 10.1186/s12951-023-02127-3

**Published:** 2023-10-06

**Authors:** Yawei Zhang, Liang Zhao, Yaocheng Li, Shuangshuang Wan, Zhiyao Yuan, Guangyue Zu, Fei Peng, Xianguang Ding

**Affiliations:** 1 State Key Laboratory of Organic Electronics and Information Displays & Jiangsu Key Laboratory for Biosensors, Institute of Advanced Materials (IAM), Nanjing University of Posts and Telecommunications, Nanjing, 210023 China; 2 Department of Periodontology, Nanjing Stomatological Hospital, Affiliated Hospital of Medical School, Nanjing University, Nanjing, 210008 China; 3 CAS Key Laboratory of Nano-Bio Interface, Suzhou Institute of Nano-Tech and Nano-Bionics, Chinese Academy of Sciences, Suzhou, 215123 China; 4 Wellman Center for Photomedicine, Massachusetts General Hospital, Harvard Medical School, Charlestown, MA 02114 USA

**Keywords:** Extracellular vesicle, Bioinformatic nanomaterials, Enrichment, Decoding, Clinical diagnostics

## Abstract

Extracellular vesicles (EVs) are membrane nanoarchitectures generated by cells that carry a variety of biomolecules, including DNA, RNA, proteins and metabolites. These characteristics make them attractive as circulating bioinformatic nanocabinets for liquid biopsy. Recent advances on EV biology and biogenesis demonstrate that EVs serve as highly important cellular surrogates involved in a wide range of diseases, opening up new frontiers for modern diagnostics. However, inefficient methods for EV enrichment, as well as low sensitivity of EV bioinformatic decoding technologies, hinder the use of EV nanocabinet for clinical diagnosis. To overcome these challenges, new EV nanotechnology is being actively developed to promote the clinical translation of EV diagnostics. This article aims to present the emerging enrichment strategies and bioinformatic decoding platforms for EV analysis, and their applications as bioinformatic nanomaterials in clinical settings.

## Introduction

As important cellular surrogates, extracellular vesicles (EVs) are increasingly recognized as attractive biomarkers for disease diagnosis. With the size range of nanoscale (50–200 nm), EVs are widely present in a variety of body fluids, such as blood, urine, saliva, and milk [1]. Recent advances in EV biogenesis demonstrated that EVs play an important role in intercellular communication and participate in the regulation of a series of physiological and pathological processes [2, 3]. They can be secreted by various cell types in human body (including endothelial cells, immune cells, platelets, and smooth muscle cells), which contain a variety of biological information, such as protein, lipids, and genetic materials (miRNA, mRNA, DNA molecules, and long non-coding RNA, etc.) [4]. These unique characteristics of EVs make them highly valuable bioinformatic nanomaterials, representing new avenues as cellular surrogates to molecular evaluation of their origin.

EVs offer multiple advantages as circulating bioinformatic nanomaterials in biofluids for bioassay. A single EV can carry comprehensive bioinformation of the origins, and therefore can be detected without tissue or other invasive samples, but instead only requires a collection of biological fluids such as blood, urine, saliva or cerebrospinal fluid to conduct the assay [5]. Unlike traditional molecular markers, EV detection can provide more accurate and refined information, especially in early diagnosis and disease monitoring due to their constant secretion by parent cells. Besides, EVs can naturally bypass the blood-brain barrier, which gives them advantages in detecting central nervous system diseases. Compared to the clinical gold standard of tissue biopsy for cancer detection, EVs-based liquid biopsy is a minimally invasive method, avoiding invasiveness and enabling multiple sampling for monitoring purposes [6].

While EVs can be identified by multiple dimensions, such as size, diameter, density, inclusion, antigen expression, concentration, etc [7–10]. EV bioinformatics remains a significant challenge for clinical diagnostics, which severely impedes basic research and clinical development: (a) improvements must be made in the isolation and enrichment of EVs. The existing approaches usually require a large amount of fluidic volume to harvest EVs and involve complex operation processes with low yields. (b) it is challenging to detect EVs with current bioinformatic decoding strategies and analytic platforms due to the relatively low abundance of molecules enriched in the nanocabinet. With the deepening understanding of EVs and the development of nanotechnology, a series of enrichment and detection technologies have been developed for EV bioinformatic decoding.

**Fig. 1:**
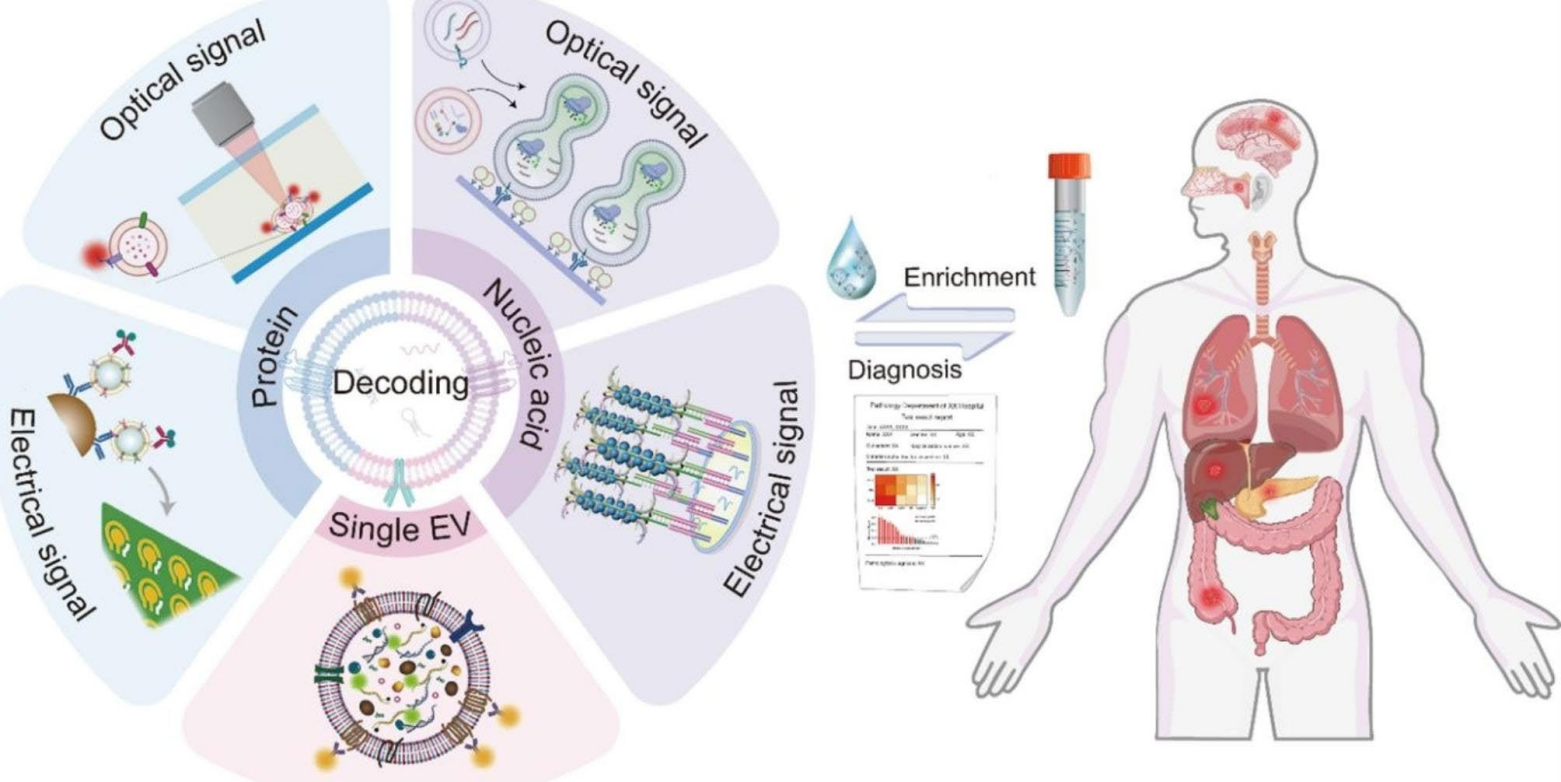
Schematic illustration of EVs as bioinformatic nanomaterials enriched from biofluids. By decoding vesicular nucleic acids or protein information through continuously updated detection platforms, various types of diseases might be diagnosed

In this paper, our review focuses on the recent advances of EVs as bioinformatic nanomaterials, ranging from characterizations, newly developed enrichment strategies, and bioinformatic decoding, to their extensive clinical diagnostic applications (Fig. 1).

## Physical characterization of EV

To evaluate the size, concentration and characteristics of EVs, a variety of optical and non-optical methods have been adapted [11–15]; however, optimization and standardization of technology for EV characterization remain essential tasks. This section will introduce some of the most popular mainstream technologies. Both of their advantages and disadvantages were analyzed.

### Microscope-based Method

Microscopes have been widely used to measure the physical characteristics of EVs [16]. Compared with ordinary optical microscopes, transmission electron microscopes and scanning electron microscopes can provide images with higher resolution to show the physical characteristics of EVs. The atomic force microscope can provide better surface topology information and microenvironment characteristics. At present, the powerful tool for evaluating the morphology of EVs is the low-temperature electron microscope, which can maintain the nearly natural state of the membrane and better observe EVs [17–20]. However, the microscope technology needs professional operation and careful sample preparation, so the specific research requirements and limitations should be considered when selecting the relevant microscope technology.

### Particle size analysis (NTA)

Nanoparticle tracking analysis (NTA) is a method of particle size determination using light scattering to track the Brownian motion of many individual particles in a liquid [21]. NTA is suitable for detecting particles with a diameter of about 10 to 1000 nanometres, which makes it an ideal choice for detecting EVs [22]. However, this technique is limited by the number of particles analyzed simultaneously. The presence of variables can also affect the measurement results, including camera level, detection threshold, viscosity, temperature, and sample dilution [23].

### Dynamic light scattering technique

Dynamic light scattering (DLS) is a technology used to detect particles’ Brownian motion or diffusion behavior. The mass of particles is measured by detecting the drift velocity of particles in solution. This method is simple to use, and does not need extra treatment of extracellular vesicles, so it will not affect the properties of the sample. However, it should be noted that it is not sensitive to the detection of small particles, and it is only suitable for small samples, so the data interpretation is complicated [13, 24].

### Tunable resistance pulse sensing

Tunable resistance pulse sensing (TRPS) is an emerging analytical technique that can be used in place of NTA to measure the concentration and size distribution of EVs [25, 26]. The TRPS monitors the current flowing through the tunable nanopores. The particle passing through the pore will cause the instantaneous change of ion current, directly proportional to the particle size. TPRS has a very high detection limit, which allows high-precision measurement of each particle in the range of 40 nanometers to 20 microns, and can provide the size information of extracellular vesicles and other parameters such as charge, concentration and concentration distribution, and the measurement is not affected by optical characteristics. However, it requires professional equipment and skills, and its operation is more complicated.

### Small Angle X-Ray Scattering (SAXS)

SAXS is a new technology for detecting the size distribution of electric vehicles. Its working principle is based on small-angle elastic scattering of X-ray photons at sample points. This technology has been widely used to study submicron solid or liquid structures [27, 28]. Compared with other technologies, SAXS is a non-destructive technology, which does not require special treatment or labeling of samples, so it will not interfere with the characteristics of samples. This enables it to maintain the original properties of the sample. In addition, SAXS has the ability of real-time measurement, which can provide high-resolution information, including data on the size, shape and arrangement of extracellular vesicles. However, it should be noted that SAXS requires very high sample preparation and stability, and must be measured in a vacuum or low humidity environment. In addition, SAXS needs special instruments, which is expensive and needs experienced professionals to operate. At the same time, radiation protection measures need to be taken during use to ensure the safety of operators.

## EV enrichment

The difficulties to be faced in the enrichment of EV are size, source and molecular composition. Besides its inherent heterogeneity, ultracentrifugation is the gold standard for the separation and enrichment of EV. However, it needs to meet the conditions of clinical application, which requires a relatively large initial sample size and expensive ultracentrifuge. Other methods, such as ultrafiltration, polymer sedimentation and molecular exclusion, have their own limitations and shortcomings, so there is an urgent need for efficient, convenient, and high-purity methods for the separation and enrichment of EV [29, 30].

### Improved enrichment methods

Numerous new EV enrichment methods have been developed to address the limitations of existing methods, such as lengthy separation times, the need for expensive equipment, low efficiency, and low purity [31]. While many of these new methods have successfully improved certain aspects of EV enrichment, a more comprehensive solution is needed to address the most challenging issues [32]. Here we summarize some recent developments.

#### Ultrafiltration

Ultrafiltration, as a potential EVs enrichment method, is currently used to enrich EVs from a large number of cells in a specific conditioned medium. The main reason ultrafiltration is not as good as ultracentrifugation is that ultrafiltration cannot avoid protein contamination. The simplest way to solve this problem is to combine ultrafiltration with other methods to Remove impurities. Glenn Vergauwen et al. studied by density gradient ultracentrifugation to quantify protein contaminants in EVs and concluded that size exclusion chromatography could effectively remove protein impurities in EVs enriched by ultrafiltration [33]. After that, in the research of Birke J et al., the operation flow of ultrafiltration combined with size exclusion chromatography is described in detail [34]. The results show that the yield and purity of EV obtained by this method are better than those by ultracentrifugation.

In addition, Chen et al. recently improved the membrane configuration of ultrafiltration and designed a new ultrafiltration strategy to combine with other methods [35]. Double-coupled harmonic oscillation was introduced into the dual-membrane filter configuration, and ultra-efficient purification of EVs was realized through negative pressure oscillation and membrane vibration activated by the double-coupled harmonic oscillator. This technique is highly effective at removing approximately 99% of protein impurities. It solves the problems of blocking, protein pollution, and processing difficulties that can arise when using ultrafiltration.

#### Immunoaffinity enrichment

An ideal immunoaffinity material should possess basic characteristics such as fast treatment time, low nonspecific adhesion, high capture efficiency, and easy release of captured EVs. However, traditional immunoaffinity enrichment techniques are limited due to poor magnetic bead stability, low enrichment efficiency, and potential impact on the biological activity of EVs [29]. Improving immunoaffinity materials represents the main approach for optimizing this method.

Nan He et al. have proposed a variety of new three-dimensional structure nano graphene immunomagnetic particles [36]. Compared with the existing EV separation based on immunomagnetic beads, this method can capture and release complete EVs as needed, improving specificity and sensitivity.

Moreover, Zhang et al. proposed a new immune material, Tim4 antibody grafted organic framework immunoaffinity tablet with strong hydrophilic metal, which can enrich EVs well under neutral conditions and retain their activity [37]. To avoid the influence of pH value change on EV activity, Tim4 @ ILI-01 immunoaffinity material captures intact EVs without damage, which can significantly help subsequent clinical diagnosis and treatment. Furthermore, the latest research by Ziyan Li et al. proposed a 2D flexible nanostructure, leveraging 2D flexible Fe_3_O_4_ − MoS_2_ nanostructures to recognize EV through multidentate affinity binding and feasible magnetic separation [38]. This method enhances the EV capture performance with both yield and separation time, affording high sensitivity. The sensitivity of this method is significantly higher than that of other nanoparticles.

#### Microfluidic

Microfluidic technology is commonly used to enrich EVs using immunoaffinity enrichment. For example, Zhao et al. recently proposed a method of EVs separation and detection based on microfluidic devices, which realized microsphere-mediated dielectrophoresis separation and immunoaffinity detection [39]. The detection range ranged from 1.4 × 10^3^ to 1.4 × 10^8^ per milliliter of EVs, and the detection limit was 193 EVs per milliliter. The addition of microfluidic can Greatly improve the efficiency, sensitivity and detection limit of immune affinity.

Based on immunoaffinity, Zhang et al. combined with electrochemistry, proposed an electrochemical microaptamer sensor, and established a sensitive, specific and reliable electrochemical microaptamer sensor for detecting EVs [40]. Through the combination of microfluidic and immunoaffinity enrichment, electrochemical analysis was added to detect EVs specifically and sensitively.

The enrichment efficiency of microfluidic chips can also be improved by changing the pattern and material of the microfluidic chip. Zhang et al. have developed a microfluidic chip integrated with three-dimensional nano-porous micropores by micro-patterning carbon nanotube (CNT) columns in microchannels [41]. The micro-scale mass transfer of biological particles can be effectively promoted by using this microfluidic chip, the nano-HB chip provides an extremely low detection limit of 10 EVs/µl.

In addition, Liang Zhao et al. proposed an automatic centrifugal microfluidic disk system combined with functional membrane [42]. The characteristic is obtaining high-quality EVs from trace blood samples within 8 min, achieving one-step separation and purification. In short, microfluidic chips can achieve efficient automated strategies to enrich EVs. A comparison of all enrichment methods is summarized in Table 1.

**Table 1:** Comparison of improved enrichment methods

Method	Improvement points	Advantages	Disadvantages
Ultrafiltration	1.Combined size exclusion chromatography 2.Double coupled harmonic oscillation	1.Solve protein pollution 2.High stability, purity	1.More instruments and longer time 2.Complex equipment
Immunoaffinity enrichment	1.Unique 3D nanoscale cavity 2.Organic framework immunoaffinity sheet 3.2D flexible nanostructure	1.Higher specificity, sensitivity. 2.Avoid the influence of pH value 3.Efficient and rapid separation and enrichment	1.High cost 2.Limited capture type
Microfluidic	1. Dielectrophoresis separation and immunoaffinity detection. 2. Electrochemical microaptamer sensor 3. Micro-patterning carbon nanotube columns 4. Automated centrifugal microfluidic disc system	1.Higher efficiency, sensitivity and detection limit 2.Sensitive, specific and reliable 3.Higher efficiency and yield 4.Automatic separation and purification	

## EV bioinformatic decoding

EVs are an emerging source of disease biomarkers and are considered one of the most promising liquid biopsies. However, the small size of EVs (< 1000 nm) and the complexity of their sources and formation factors pose significant challenges to extracting clinical information using existing methods. Traditional detection methods of protein and RNA, such as Western blotting and RT-qPCR, require large sample sizes and can only accurately reflect the information of the detected sample, rather than allowing for differential analysis of EVs with different characteristics [43]. Therefore, new bioinformatic decoding methods are urgently needed to obtain more comprehensive information on EVs.

### Protein bioinformatic decoding

Proteins are essential components of human cells and tissues and participate in many life activities. Analyzing and detecting proteins can provide insights into the state of cells and tissues. By conducting high-throughput proteomic analysis of EVs, we can better understand the biological load and function of EVs [12, 44]. This understanding can help provide new insights into complex changes in cancer and potentially provide personalized treatment options for patients.

### Improved protein decoding method

Conventional EV protein analysis methods, for example, Western blot and enzyme-linked immunosorbent assay (ELISA), require a large number of samples and cannot quantify rare molecular markers. Clinical use of these methods is not suitable for large cohorts of patients [45]. Here are some new EV protein detection methods developed in recent years.

#### Flow Cytometry

Flow cytometry (FCM) is a widely used tool in basic and clinical research for rapidly analyzing single-cell surface proteins. However, the particle size detected by traditional FCM is much larger than that of single EVs, making this method unsuitable for analysis of single EVs. At present, there are two ways to solve this problem. One way is to change the FCM instrument to improve detection sensitivity. Aizea Morales-Kastresana et al. proposed new EV staining and EV analysis methods, which provide a basis for further development of nanoflow cytometry and other high-resolution cytometry methods [46].

Another way is to expand the total size of EVs to the detectable size range of conventional FCM. The typical method is to use microspheres to load EVs for FCM analysis. But high abundance of free proteins in clinical samples may also be nonspecifically adsorbed on microspheres, resulting in false positive results and decreased sensitivity. Liu et al. proposed a pH-mediated EV assembly method, which enabled EV to be directly analyzed by traditional flow cytometry (FCM) [47]. To control the surface wettability of EV, pH-responsive diacyl lipid conjugated polymers were fitted into EV membranes. Regulate pH value to form EV clusters. The cluster size (1 μm) is larger than the detectable size of conventional FCM, so conventional FCM can be used to analyze proteins in EV.

#### Fluorescence imaging

Reproduced with permission from Chao Liu et al [52]. Copyright Nature Publishing Group (2019). Reproduced with permission from Fei Tian et al [12]. Copyright Nature Publishing Group (2021).

At present, fluorescence imaging is widely used in clinical medicine, and it has also made significant progress in detecting EV proteins. Vojtech Vinduska et al. proposed a method for detecting EV surface proteins using quantum dots combined with immunomagnetic capture and enrichment [48]. This method can detect different surface protein markers on EVs of varying cell lines specifically and quantitatively. There is no need for a large number of plasma sample pretreatment and complicated and expensive instruments. Detection is carried out by volume fluorescence measurement of conventional fluorescence spectrometer. In addition, Rongrong Huang et al. developed a fluorescence assay for EV based on branched rolling circle amplification (BRCA) [49]. This method has high specificity for target EV proteins. It is a rapid and cheap method for detecting EV marker proteins.

Furthermore, Zhang et al. developed a local fluorescence imaging method to detect the digital map of protein on a single EV with ultra-sensitivity [50]. This method can analyze proteins from individual EVs of 30 µL diluted plasma without a purification step. This method also saves the filtration and purification steps, can detect a single EV membrane protein, and obtain EV protein data with high accuracy.

#### Thermophoresis sensor

Thermophoresis is an unlabeled technique for nanoparticles precisely in a temperature gradient. As a means of effectively enriching particles in liquid, thermophoresis is widely used in EV detection. Yike Li et al. proposed a thermophoresis-mediated DNA computing device for EV detection [51]. This method can specifically and sensitively recognize protein biomarkers on EV surface by thermophoresis-mediated DNA logic device.

**Fig. 2:**
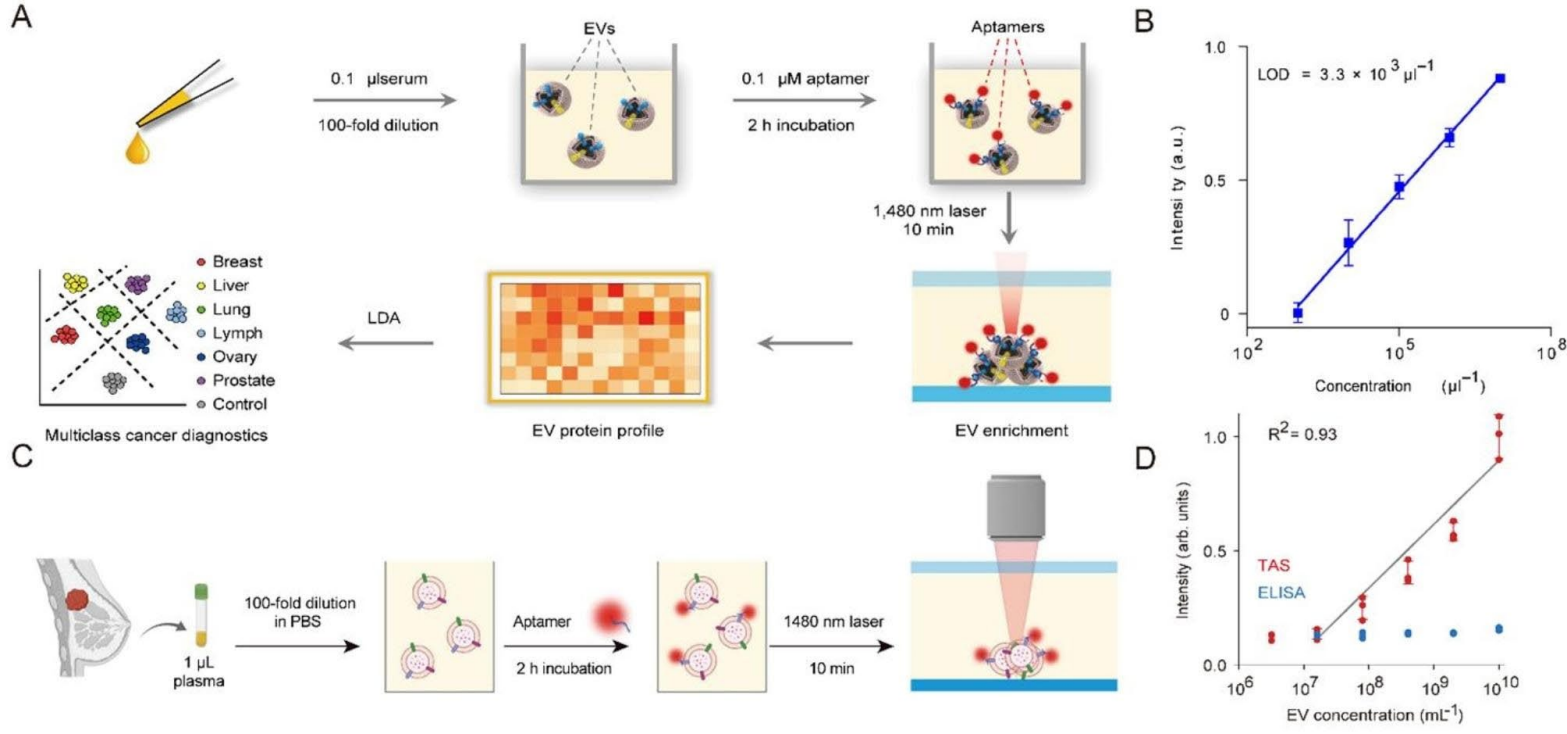
Thermophoresis Sensor. (**A**) Schematic of the TAS procedure. Using a panel of 7 aptamers, the protein signatures of EVs in 0.1 μl human serum were profiled by TAS. (**B**) Sensitivity of TAS for the detection of HepG2 EVs. (**C**) Schematic of the TAS procedure. Amplify the fluorescence signal of aptamer-bound EVs, enabling rapid and sensitive detection of EV protein markers. (**D**) Sensitivity Comparison between TAS and ELISA.

The characteristics of thermophoresis sensors do not need to pre-process samples, which is an excellent advantage in detecting EV proteins that require separate and complex processing. Moreover, Chao Liu et al. proposed a thermophoresis aptamer sensor that used aptamer to enrich EV (Fig. 2A) [52]. Aptamer-bound EVs produce amplified fluorescence signals during accumulation, and their intensity is related to the expression level of EV’s target surface proteins (Fig. 2B). This detection method is low-cost, rapid, accurate and requires a small serum volume (< 1 µl). It takes less than 3 h for TAS to detect seven EV protein markers in a serum sample, and diseases can be effectively distinguished by the LDA algorithm.

Furthermore, Fei Tian et al. developed a method. It eliminates the influence of pollution and uses a machine learning algorithm to establish a direct, sensitive and economical thermoelectric aptamer sensor [12]. In this method, aptamers are combined with thermophoresis enrichment of EVs to generate amplified fluorescence signals, and the expression level of EV surface proteins is judged by signal intensities (Fig. 2 C–D). This method can directly use a small amount of plasma detection of EV: By detecting the EV characteristics of seven protein markers, early detection and classification of six different cancer types have been achieved.

#### SPR biosensor

Among optical biosensors, nano-plasma biosensors are used in EV detection due to their excellent sensitivity. Based on identifying the subtle signal differences enhanced by strong electromagnetic fields, nano-plasma biosensor based on local surface plasmon resonance (LSPR) has considerable prospects in unlabeled quantitative analysis of EVs. Its detection limit is as low as that of a single EV [53]. EV proteins can be analyzed by refraction changes.

Reproduced with permission from Sangmoo Jeong et al [58]. Copyright American Chemical Society (2016). Reproduced with permission from Jongmin Park et al. [60]. Copyright Nature Publishing Group (2021).

Chang Liu et al. developed a compact SPR biosensor with intensity modulation, which uses a traditional SPR sensing mechanism and does not need nanostructure manufacturing, and is used to capture EVs and characterize EVs’ proteins [54]. Although this scheme sacrifices some sensitivity and resolution, the compact SPR biosensor still has better detection sensitivity than ELISA. This scheme has enough sensitivity to analyze EV proteins to distinguish diseases. This sensor can detect EV protein biomarkers sensitively, unlabeled, in real-time and cost-effectively.

In addition, Wang et al. used the unique advantages of LSPR biosensing and nano-plasma labeling to develop a nano-plasma sandwich EV immunoassay, which can quantitatively detect EVs and accurately analyze EV protein (PD-L1) in a single assay [55]. This method realizes high sensitivity quantification of EVs and accurate identification of EVs subclasses.

Besides, Huiwen Xiong et al. proposed an ECL immunosensor based on local surface plasmon resonance (LSPR) between AUNP and PDOT [56]. It can be used to determine EV surface protein markers. The authors use polymer dots, which are easy to synthesize, easy to modify, and have stable electrochemical and optical properties of electrochemiluminescence (ECL) luminescence. The sandwich ECL immunosensor has high stability and repeatability. ECL immunosensor based on LSPR realizes sensitive multiple EV protein map, which can distinguish different types of cancer and distinguish cancer from normal group. Moreover, this universal platform can evaluate EVs’ surface protein expression levels from various cell lines.

#### Integrated magnetic-electronic EV (iMEX) sensor

The iMEX sensor utilizes an integrated magnetic electrochemical analysis to isolate and analyze EVs from patient samples. Unlike traditional methods that require filtration or centrifugation, iMEX can directly capture cell-specific EVs from complex media. Additionally, the sensor features magnetic enrichment and enzyme amplification, which enable high detection sensitivity. The electrochemical detection scheme further enhances the sensor’s detection sensitivity, making it a versatile tool for a wide range of applications. Moreover, the sensor’s compact size allows miniaturization, further expanding its potential applications [57].

**Fig. 3:**
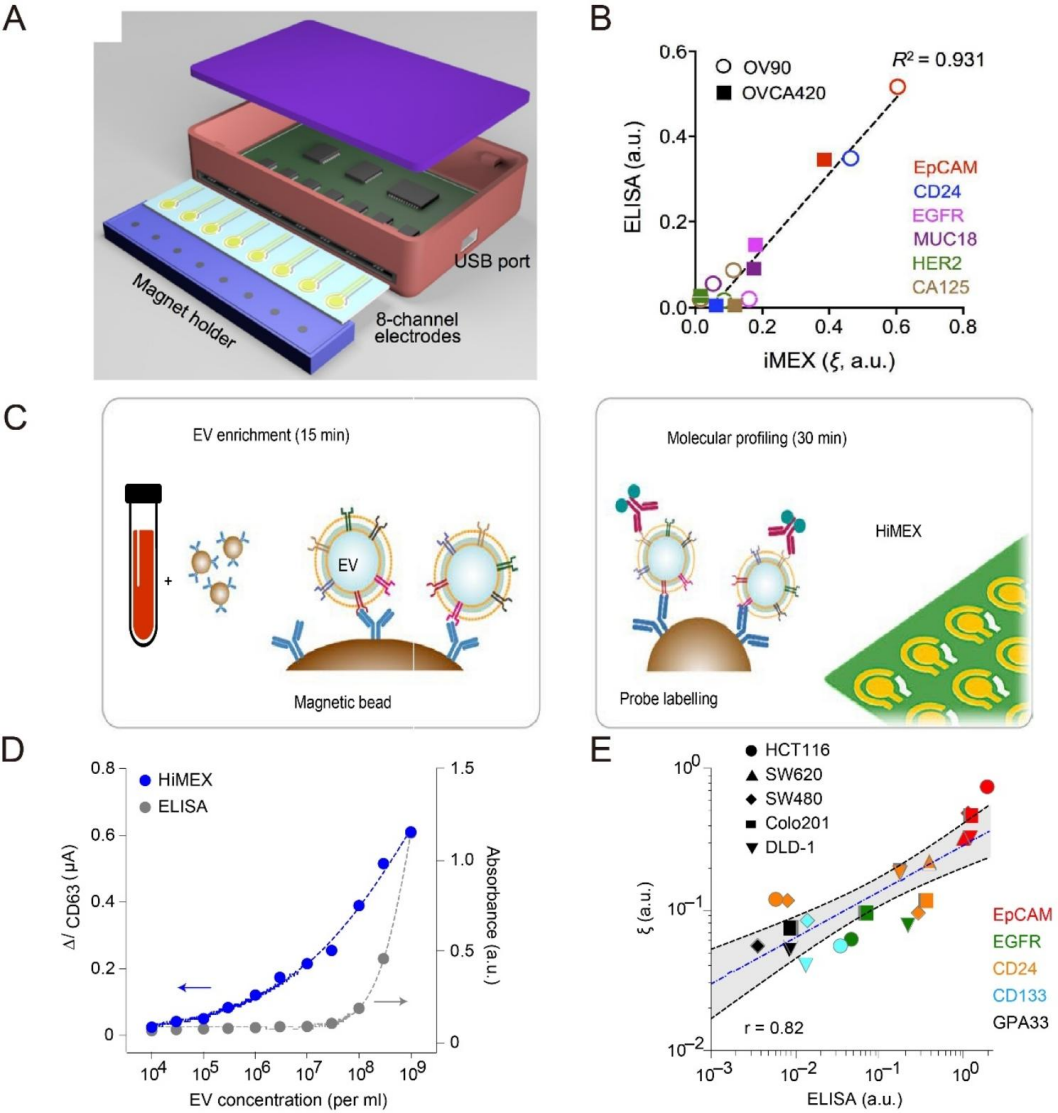
iMEX Sensor. (**A**) Sensor schematic. The sensor can simultaneously measure signals from eight electrodes. (**B**) Comparison between iMEX and ELISA. (**C**) Two-step HiMEX assay protocol. (**D**) The HiMEX assay had superior sensitivity and a more comprehensive dynamic range than (ELISA). (**E**) The HiMEX results showed a good match with those from ELISA.

Sangmoo Jeong et al. has developed a portable eight-channel device capable of analyzing multiple protein markers simultaneously in one hour, offering superior analysis sensitivity and speed compared to traditional methods [58]. By combining magnetic enrichment and enzyme amplification, this method enables high sensitivity and cell-specific EV protein detection (Fig. 3A). The iMEX was compared to the ELISA and showed a high correlation between the two methods. iMEX analysis is faster and consumes fewer samples than ELISA, making it suitable for practical clinical applications (Fig. 3B).

In addition, Yu An et al. have developed a magnetic dielectric chemical sensor for analyzing protein labeling information in EVs derived from breast cancer cells [59]. This sensor can accurately and sensitively detect four tumor marker proteins on the EV surface of different breast cancers.

Furthermore, Jongmin Park et al. have introduced a high-throughput iMEX method called HIMEX, which uses 96-well analysis and integrates antibody-coated magnetic beads to enrich EVs (Fig. 3 C–E) [60]. Electrochemical detection of EVs after enzyme amplification can be done in less than an hour. All iMEX devices are portable, and the detection and analysis process is short, with results usually obtained within an hour, showing great potential for clinical applications.

#### Electrochemical sensor

Electricity plays an essential role in living organisms, providing valuable insights into cell metabolism, nerve impulse transmission, and cell membrane structure and function through electrochemical detection. Tugba Kilic et al. have introduced a rapid and multi-channel method for EV protein profiling analysis called iPEX (EV Impedance Spectroscopy) system [61]. This approach significantly shortens the EV protein analysis process and offers high sensitivity by enabling the detection of multiple EV protein markers simultaneously on a single chip. Besides, Lee et al. have proposed a straightforward and sensitive electrochemical method for detecting EV surface protein nanoparticles [62]. This method utilizes gold nanoparticles (AuNP) functionalized with two types of antibodies to generate redox currents proportional to the EV concentration and EV surface marker expression levels. By using electrochemical readings, this method detects multiple EV surface markers with a single electrode.

Furthermore, Ayemeh et al. have developed an ultra-sensitive electrochemical aptamer sensor for multiple detections of biomarkers in EVs based on the electrochemical signals of metal ions [63]. This method employs various composite materials to modify the screen-printed carbon electrode, which improves the electrochemical signal. By measuring the differential pulse voltammetry signals of these metal ions, multiple EV proteins can be analyzed simultaneously. This technique provides high sensitivity, high selectivity, wide linear range, and low detection limit for target EVs. A comparison of all protein decoding methods is summarized in Table 2.

**Table 2:** Comparison of improved protein decoding methods

Method	Improvement points	Advantages	Disadvantages
Flow cytometry	1.Nanoflow cytometry 2.form EV clusters,size larger than the detectable size of FCM	1.Increase detection limit 2.High stability, no blockage, no protein pollution	1.Equipment still needs to be developed 2.Complex equipment
Fluorescence imaging	1.Fluorescence spectrometry with quantum dots 2.Branched rolling circle amplification 3.Ring circle amplification digital detection	1.Plasma samples do not need pretreatment 2.High specificity, fast and cheap 3.Low detection limit and high accuracy	1.Limited accuracy 2.Limited capture type 3.Multi-step and long process
Thermophoresis Sensor	Thermophoresis aptamer sensor	Low cost and fast detection and Simultaneous detection of multiple marker proteins	Need better algorithms to eliminate the impact of pollution
SPR biosensor	1.Intensity-modulated, compact SPR biosensor 2.nanoplasmonic EV immunoassay utilizing 3.ECL immunosensor	1.Label-free, real-time and cost-effective detection 2.High sensitivity and accuracy 3.Simultaneous detection of multiple EV surface proteins	1 Expense of sensitivity and accuracy 2.Only one protein can be detected at a time 3.Only surface protein can be detected
Integrated Magnetic- Electrochemical EV(iMEX) Sensor	Integrated multi-channel separation and detection	1.Device portability 2.High throughput analysis and detection	1.Poor versatility of equipment
Electrochemical sensor	1.iPEX (impedance profiling of EV) 2.Anoparticle-Enabled Multiplexed electrochemical Immunoassay 3.Ultrasensitive electrochemical aptasensor	1.Rapid simultaneous detection of multiple proteins 2.Simple detection with high sensitivity 3.High specificity, wide linear range	1.Only surface protein can be detected 2.Only surface protein can be detected 3.Complex materials

### Nucleic acid bioinformatic decoding

EVs are rich in both nucleic acids and proteins. Their protective membrane makes EVs’ miRNA molecules more stable than free miRNA. In some cases, EVs also carry short fragments of DNA that represent the genomic DNA of parent tumor cells, making it possible to identify mutations. However, detecting these nucleic acids is challenging using traditional methods, due to the small amounts present in EVs [64]. Thus, developing efficient and sensitive detection methods is crucial.

### Improved nucleic acid detection methods

Nucleic acids contained in EVs carry a variety of information. If it can be fully detected and studied, it is expected to be used to detect many complex clinical diseases [65]. Currently, the diagnosis and detection of nucleic acids contained in EVs have considerable potential, and the demand for EV nucleic acid diagnosis and detection continues to increase. The development of new analysis and detection technologies is also continuously promoted to achieve more efficient and rapid EV nucleic acid extraction and analysis.

#### Liposome fusion

Reproduced with permission from Bo Ning et al [67]. Copyright Nature Publishing Group (2021).

Liposome fusion is a crucial biological process characterized by selective recognition mechanisms for molecular and ionic cargo transport. Imitating the natural membrane fusion mechanism has great potential for developing biosensors, particularly for amplification detection. In the work of Coline Jumeaux et al., sequence-specific DNA-mediated liposome fusion was used to detect miRNA with high selectivity [66]. By using a fluorescence output based on FRET, miR-29a could be detected sensitively and specifically at a nanomolar concentration within 30 min, with a detection limit of 18 nm.

**Fig. 4:**
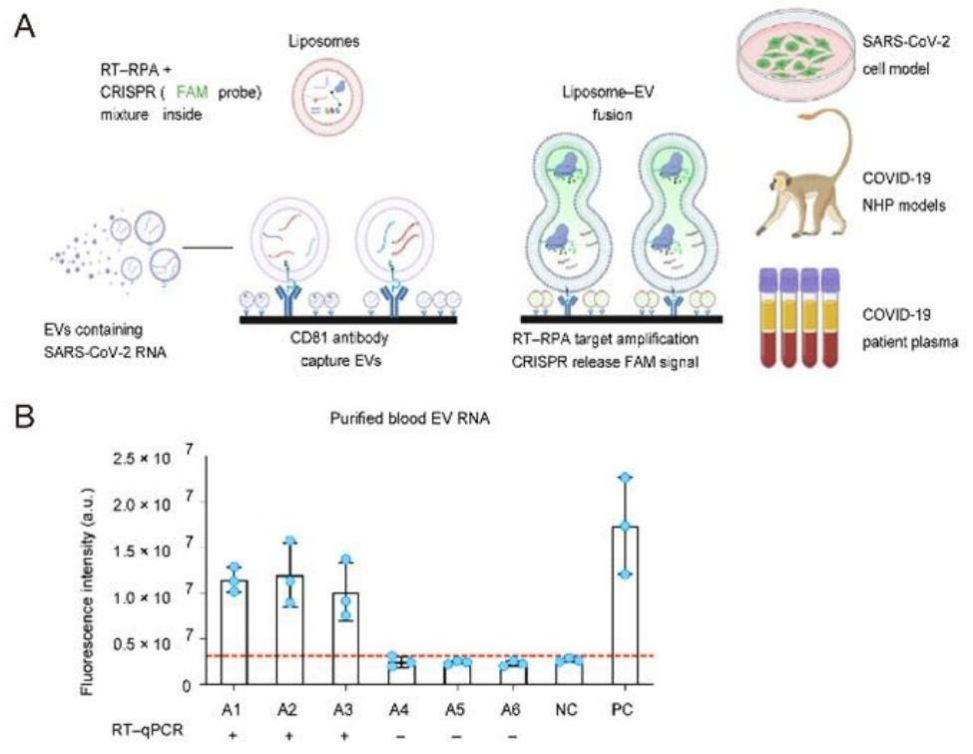
Liposome fusion detection. (**A**) Schematic of the liposome fusion detection. EVs fusion with RT–RPA–CRISPR-loaded liposomes. (**B**) Accurate results compared to qPCR.

In addition, Bo Ning et al. proposed a detection method that can rapidly, efficiently, and with high throughput detect EV miRNAs within two hours by using a fusion vesicle that simulates a virus [67]. Combined with flow cytometry, it can detect EV miRNA by fusing with simulated virus membranes and using liposomes to fuse EV membranes with liposome membranes. Furthermore, Bo Ning et al. proposed a detection method that directly captures EVs from plasma and fuses them with reagent-loaded liposomes to detect nucleic acid targets (Fig. 4A–B). This method accurately identified COVID-19 patients, including challenging cases missed by RT-qPCR. Immunoaffinity is used to enrich EVs, enabling direct detection from plasma and improving efficiency.

#### SERS sensor

Surface-enhanced Raman scattering (SERS) is a promising technique for biomolecular analysis due to its quick response time and superior sensitivity. One common approach to improve the sensitivity of SERS sensors is to create a “hot spot” structure that enhances the local electromagnetic field strength. However, applying SERS sensors for detecting miRNA in extracellular vesicles (EVs) in complex samples remains challenging [68].

To address this challenge, Zhao et al. proposed a microfluidic platform that integrates EV enrichment, subsequent lysis, and miRNA detection into a single device [69]. The microfluidic chip contains a magnetic enrichment chamber, a serpentine fluid mixer, and a plasma surface-enhanced Raman substrate with a trapping probe. Upon miRNA release, the capture probe activates and triggers rolling circle amplification (RCA) to generate multiple “hot spots” that enhance the SERS signal. This SERS sensor dramatically improves sensitivity and increases the limit of detection (LOD) for EV miRNA to 1 pmol/L.

Additionally, Jiang et al. proposed a new method based on SERS and magnetic nanoparticles for the in-situ detection of microRNAs in secretions [70]. This method selectively detects target miRNAs using a SERS detection method based on targeted aptamers, thus avoiding interference from other miRNAs. The method can detect very low concentrations of miRNA in complex biological samples with high selectivity and sensitivity. This technique has promising applications in biomarker detection and molecular diagnosis.

#### Electrochemical sensor

Electrochemical methods are also widely used in nucleic acid detection. Liu et al. enzyme-free electrochemical biosensor based on local DNA cascade displacement reaction and multifunctional DNA nanosheets is used for ultra-sensitive detection of EV miRNA [70]. The sensing system only contains nucleic acid, does not involve protease and inorganic nano-materials, and realizes simple operation steps.

In addition, Zhang et al. proposed a new electrochemical biosensor based on a catalytic hairpin assembly circuit for EV miRNA detection [71]. The formed hypercondensate can provide a greatly enhanced signal-to-noise ratio, with a linear range from 10 fm to 100 nm and a detection limit of 7.94 fm. This method is a rapid, portable, disposable, enzyme-free isothermal method to detect EV miRNA with high sensitivity and specificity.

Furthermore, Zhang et al. used a fast electrochemical biosensor assembled by multifunctional DNA tetrahedron-assisted catalytic hairpin to detect EV miRNA [72]. This method can improve the local concentration and the efficiency of enzyme-free amplification. Therefore, ultra-sensitive detection can be realized in a short time. Compared with qRT-PCR, electrochemical biosensor has the advantages of enzyme-free, isothermal operation, simple probe design and low cost.

#### Thermophoresis sensor

Thermophoresis also has good application in detecting nucleic acids in EV. Junxiang Zhao et al. proposed a thermophoresis sensor using NanoFlares that can perform in situ detection of miRNAs in EV without the need for RNA extraction or target amplification [73]. This method exhibits a good linear response to EV miRNA. The detection limit is 0.36 fM.

Furthermore, Ziwei Han et al. developed an ultra-sensitive detection method that combines FDT detection and thermophoresis enrichment for in situ measurement of EV miRNA. This DNA tetrahedron-based thermophoresis assay is simple and can quantitatively detect the ultra-low concentration of mRNA in serum EVs [74]. Comparison of all nucleic acid decoding methods is summarized in Table 3.

**Table 3:** Comparison of improved nucleic acid decoding methods

Method	Improvement points	Advantages	Disadvantages
liposome fusion	1.Sequence specific DNA-mediated liposome fusion 2.Virus-mimicking fusogenic vesicle 3.Antibody-mediated capture and liposome-mediated reagent delivery	1.Fast, sensitive and specific 2.Rapid detection of miRNA 3.High accuracy	Need more stable membrane fusion
SERS sensor	1.Microfluidic surface-enhanced Raman scattering 2.Target-triggered hot-spot SERS strategy	1.Low sample consumption and high accuracy 2.High sensitivity in situ detection	1.Cannot detect multiple targets at the same time 2.Need suitable substrate
Electrochemical sensor	1.Localized DNA cascade displacement reaction (L-DCDR) 2.Polymerization catalytic hairpin assembly (SP-CHA) 3.Multifunctional DNA tetrahedrons assisted catalytic hairpin assembly	1.Fast response and ultra-high sensitivity 2.Easy to operate, wide linear range 3.Ultra-sensitive detection in a short time.	2.Sample needs pretreatment 3.Slightly poor stability
Thermophoresis Sensor	1.NanoFlares 2.DNA tetrahedron	1.Situ detection 2.High sensitivity	

## Single EV bioinformatic decoding

Traditional detection techniques such as ELISA and RT-qPCR provide an overall molecular profile of EVs, but their heterogeneity poses challenges in medical diagnosis and treatment. This diversity makes it difficult to determine the physiological function, composition, and subpopulations of EVs. Therefore, analyzing single EVs is crucial. However, conventional detection methods lack sensitivity, preventing the detection of protein or mRNA expression at the single EV level. New technologies are needed to detect single EVs with high sensitivity and specificity, enabling the co-localization of different molecular markers on the same EV and obtaining frequency distributions of markers in the entire population [75]. Achieving these goals will facilitate the practical clinical application of EVs. In the following sections, we will introduce several techniques for single EV analysis.

### Flow cytometry

Reproduced with permission from Jian Zhou et al. Copyright American Association for the Advancement of Science (2020) [79]. Reproduced with permission from Zijian Yang et al. Copyright American Chemical Society (2020) [80].

Imaging flow cytometry (IFC) combines traditional flow cytometry with fluorescence microscope. The morphology and multi-parameter fluorescence of samples can be quickly analyzed at the level of single cell and population. In the initial study, imaging flow cytometry (IFCM) can be used to characterize large EVs, and it was optimized for single EVs detection in the study of André Görgens et al. by using antibody labeling method, IFCM is used to detect different EVs and single EVs subsets reliably [76]. The final results of the authors show that IFCM will help significantly improve the ability to evaluate EV heterogeneity, and help identify specific subsets of EVs as valuable biomarkers for various diseases.

Furthermore, Wouter W. Woud et al. used IFCM directly used to detect single EVs in plasma [77]. The author eliminated the selection deviation or artificial interference caused by EVs separation technology, and proposed a gating strategy to identify single EVs. According to this gating strategy, EV subsets in human plasma can be easily identified based on co-localizing two fluorescent labels bound to EV membranes. This method avoids the troublesome process of enriching EVs in plasma, and directly characterizes individual EVs.

### Fluorescence imaging

Multiple fluorescence imaging technologies have been applied to single EV detection. However, these methods have some disadvantages, such as limited signal intensity for rapid imaging of fluorescent dyes and limited light stability for long-term characterization. Huang et al. used conversion nanoparticles to quantify single EVs [78]. Semiconductor quantum dots (QDs), as a choice of single molecule probes, have been used for imaging single EVs. Using the unique optical properties of this material and the background elimination characteristics of total internal reflection fluorescence imaging technology, the heterogeneous expression of EV antigens was evaluated. The detection limit was nearly three orders of magnitude lower than standard enzyme-linked immunosorbent assay (ELISA). However, this method needs to pretreat the sample to separate EVs.

**Fig. 5:**
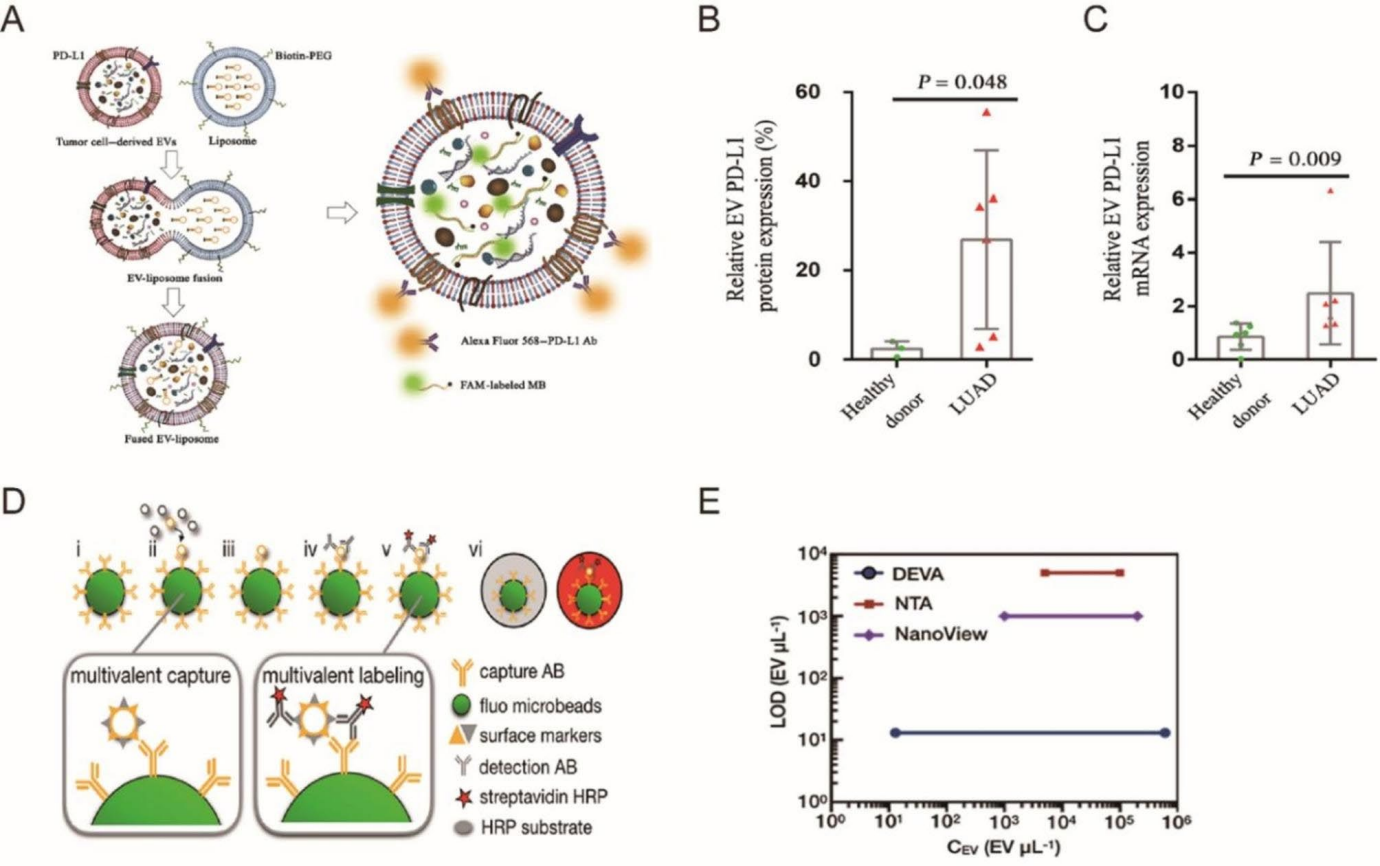
Single Extracellular Vesicle Detection. (**A**) Digital ELISA assay for single-specific EV detection. (**B**) Comparing the sensitivity and the dynamic range of DEVA, NTA, and Nanoview. (**C**) Illustration of the HNCIB system for simultaneous detection of PD-L1 membrane protein and mRNA in a single EV. (**D**) Statistic analysis of the differential membrane protein expression of PD-L1 + EVs.

Furthermore, Zhou et al. Integrated separation and fluorescence imaging into a nano-biochip (Fig. 5A–C) [79]. This method is a high-throughput nano-biochip integrated system suitable for liquid biopsy, which can simultaneously detect mRNA/miRNA protein in a single EV. This technique allows rapid single EV analysis and requires a tiny sample size. This method can be used to reliably evaluate miRNA, mRNA and protein expression in EVs in clinical samples.

### Digital analysis

Encoding nucleic acids by different means for digital analysis and detection has the advantage of obtaining a large amount of information through multiple simultaneous EV detections. Enzyme-linked immunosorbent assay (ELISA) is one of the most commonly used labeled immunoassay techniques. its sensitivity is not enough to analyze a single EV. Yang et al. developed a photo-fluid platform based on droplets. A high-throughput droplet digital enzyme-linked immunosorbent assay was ultra-sensitive to detect a single extracellular vesicle, thus realizing the analysis of EV heterogeneity [80]. A single EV was loaded into a droplet using digital droplet technology. It was encoded by bar code and applied to fluorescence detection of digital enzyme-linked immunosorbent assay (dELISA) (Fig. 5D–E).

In the study of Kaizhu Guo et al., long ssDNA was grown in situ on the surface of EVs through Roll Circle Amplification (RCA) to identify single EVs supported by DNA nanostructures [81]. This method can perform rapid and high-throughput analysis of surface proteins at individual EVs level.

## Clinical detection and application of EV

EVs are present in almost all types of cells throughout the body and in most body fluids, serving as carriers for diverse biomolecules and playing a crucial role in cell-to-cell communication [82]. The phenotype of EVs generally reflects the molecular characteristics of its derived cells, and it is a potential biomarker for clinical diagnosis [32]. Here we present various studies using EVs to detect the diagnosis and prognosis of cancer and central nervous system disorders.

### Cancer diagnosis

Studies have shown that the level and quantity of EVs in body fluids may vary significantly with disease. By analysis of EVs can help researchers to identify various diseases, including cancer and infectious diseases, at an early stage, thus promoting the clinical diagnosis and prognosis of diseases [83].

#### Respiratory diseases

Respiratory system is the general name of the organs in which the body exchanges gas with the outside world. EV is particularly useful in the normal biological function of the respiratory system and the pathogenesis of diseases [84]. The biological molecules (such as protein, lipids and genetic materials) it carries can reflect the state and nature of the host cells, which means that these EVs have the ability to reflect the disease state.

##### **Lung cancer**

Lung cancer stands as a prevalent form of cancer worldwide, with a mere 19% 5-year survival rate, and the prognosis of early lung cancer patients is better than that of late lung cancer patients. Therefore, early detection, diagnosis, and prognosis of lung cancer can improve the survival rate of patients. As a new functional biomarker for tumor diagnosis, extracellular vesicles have entered people’s field of vision [85].

Analysis and identification of EVs isolated from pleural effusions in patients with lung cancer and patients with benign inflammatory processes by Per Hydbring et al. revealed differences in expression levels of 17 microRNA and 71 mRNA in pleural effusions between the two patients [86]. This study demonstrates the utility of EV analysis in differentiating lung cancer patients from those with benign inflammatory processes, presenting a promising avenue for accurate tumor diagnosis.

In addition, Xiance et al. conducted extensive miRNA analysis of EVs in patients with early-stage non-small cell lung cancer and healthy individuals [87]. By verifying the miRNA of the related specific EVs, their results show that the related miRNA can be used as a specific biomarker for diagnosing adenocarcinoma.

##### **Nasopharyngeal carcinoma**

Nasopharyngeal carcinoma (NPC) is a type of malignancy associated with the Epstein-Barr virus (EBV), characterized by a complex etiology involving viral, environmental, and genetic factors. Extensive studies have shown that different EVs, such as EBV-associated EVs and pure nasopharyngeal carcinoma-derived EVs, isolated from the serum of patients with nasopharyngeal carcinoma, are involved in regulating gene expression in cell proliferation, differentiation and stress response [88].

Tumor-derived EVs (TEX) carry specific surface markers for primary tumor cells. Liu Wanli et al. detected tumor-derived extracellular vesicles (TEX) in blood and proved the practical utility of plasma LMP1 + and EGFR + extracellular vesicles as potential markers for early diagnosis of nasopharyngeal carcinoma [89].

MicroRNA RNA (miRNA) in EV can also be used as a biomarker for cancer diagnosis. Jiang et al. compared the miRNA spectrum of plasma-derived sEV between nasopharyngeal carcinoma patients and healthy controls [90]. It is found that many miRNA models can distinguish nasopharyngeal carcinoma patients from normal people, indicating that the plasma-derived miRNA model based on sEV can be used as an alternative or supplementary method for diagnosing nasopharyngeal carcinoma.

##### **SARS-CoV-2**

Detection of samples from the respiratory tract using RT-qPCR is the gold standard for clinical diagnosis of COVID-19. Still, it has poor overall sensitivity to novel coronavirus RNA in plasma, necessitating the search for new biomarkers to better diagnose and prognosis patients. The infected cells secrete EVs containing pathogen-derived factors that will accumulate in EVs. The severity of the disease can be measured by decoding the information contained in the EVs [91].

For example, Bo Ning et al. accurately identified COVID-19 patients by binding EVs captured in blood to reagent-loaded liposomes, including those not recognized by RT-qPCR. [67] This method can detect SARS-CoV-1 positive EVs the second day after infection, extending the signal detection duration. The method may be used to diagnose a patient without SARS-CoV-19RNA in the respiratory tract.

Reproduced with permission from Hu, J et al [95]. Copyright Nature Publishing Group (2017).

Reproduced with permission from Melo, S et al [94]. Copyright Nature Publishing Group (2015).

In addition, Giuseppe Cappellano et al. performed rapid and reliable testing of untreated blood samples to evaluate the diagnostic performance of extracellular vesicles from circulating platelets (PLT) as a biomarker of COVID-19 infection, and the results showed that PLT-EVs had a good diagnostic performance as a diagnostic biomarker of Sars-Cov-2 infection [92].

#### Digestive system diseases

The digestive system is mostly internal organs, and malignant tumors have no symptoms in the early stage, so it is difficult to find and diagnose without specific examination. Survival is generally low in patients with advanced digestive system tumors. Therefore, it is imperative to study new non-invasive and highly sensitive tumor markers to detect early tumors [93]. As EVs are widely distributed in body fluids, they are stable, easy to extract, and closely related to tumor cells. Therefore, researchers can diagnose cancer by detecting the contents of EVs.

##### **Pancreatic cancer**

Pancreatic cancer is an atypical gastrointestinal malignancy with hidden clinical symptoms, which is difficult to diagnose and treat. Early diagnosis and treatment are the keys to improving the prognosis of pancreatic cancer.

**Fig. 6:**
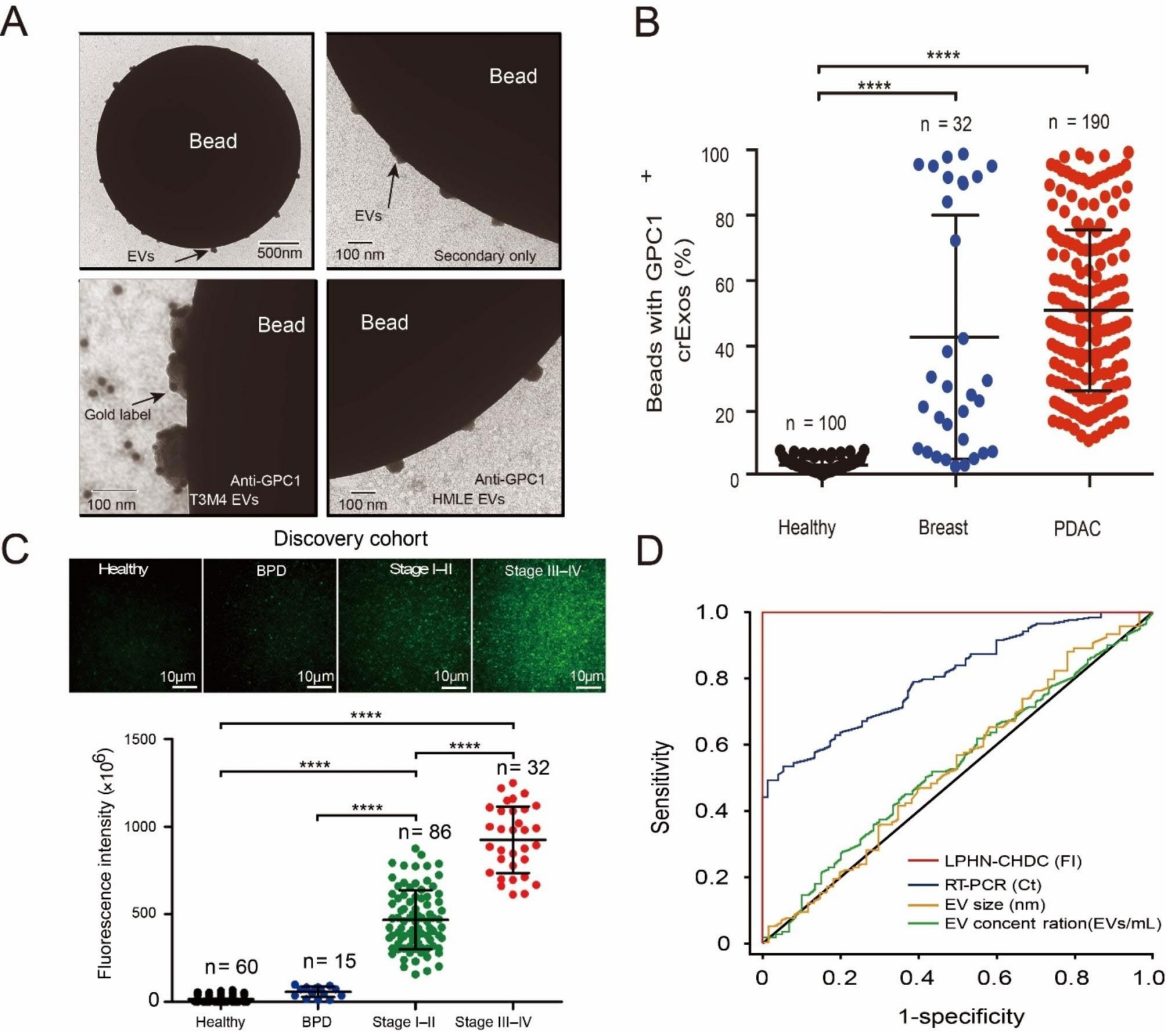
GPC1 in serum EVs and its clinical diagnostic application. (**A**) TEM of bead-bound EVs and immunogold labeling of GPC1. (**B**) Percentage of GPC11crEV beads in different patients. (**C**) GPC1 mRNA expression from different patients in the cohort was found and fluorescence intensity was calculated using METLAB. (**D**) ROC curve analysis of TIRF images

Studies have shown that the GPC1 membrane protein on the surface of EV is an effective biomarker for the detection of pancreatic cancer. Sonia A. Melo et al. isolated GPC1 + circulating EVs from the serum of cancer patients and mice(Fig. 6A) [94]. Analysis of the tests revealed higher concentrations of GPC1 + circulating EVs in patients with PDAC disease than in healthy subjects (Fig. 6B). Their results suggest that GPC1 + circulating EVs could be a potential biomarker for noninvasive diagnosis and screening for pancreatic cancer.

On this basis, Hu et al. developed a nanoparticle-based biochip for detecting GPC1mRNA in patients’ serum EVs [95]. The chip was used to detect serum of patients with pancreatic cancer and it was found that PDAC patients could be effectively distinguished from other patients and normal people (Fig. 6C) and the accuracy and sensitivity of the test results were high (Fig. 6D), indicating that the diagnosis of pancreatic cancer could be made by detecting EVs in serum.

In addition, Kai Liang et al. developed a nanoparticle-based rapid detection method for EV in plasma to detect pancreatic cancer EV, ephedrine type A receptor 2 (Ephia2) [96]. The authors compared and quantified normal and tumor-derived EVs, identified candidate biomarkers for selective enrichment of EVs in pancreatic cancer cells, and successfully used these biomarkers to differentiate pancreatic cancer patients from other patients.

Reproduced with permission from Sun, N et al [98]. Copyright Nature Publishing Group(2020).

##### **Hepatocellular Carcinoma (HCC)**

Hepatocellular carcinoma (HCC) is the major histological subtype of liver cancer, accounting for 90% of primary liver cancer. The high recurrence rate and high metastasis rate of liver cancer lead to its poor prognosis. Transplantation is the most effective treatment for liver cancer. However, in the process of transplantation, the recurrence rate and metastasis rate of tumor are very high [97]. Therefore, early detection of liver cancer is crucial to reduce the high mortality rate of liver cancer.

**Fig. 7:**
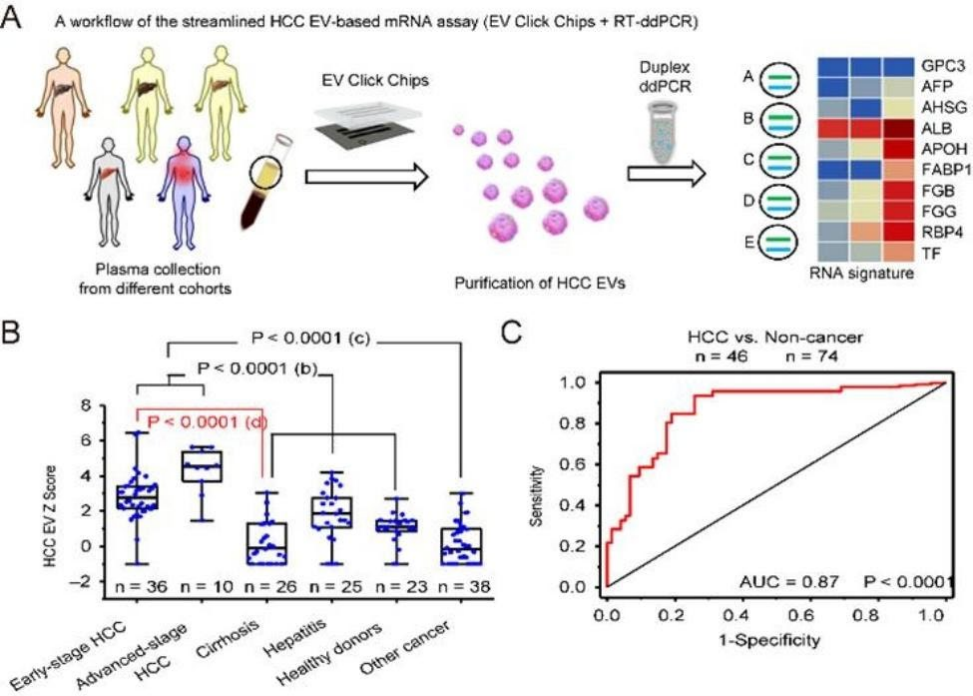
Workflow of HCC EV purification system and early detection of HCC use numerical scoring. (**A**) HCC EV purification system workflow followed by quantification of HCC-specific transcript in purified EVs. (**B**) HCC EV Z score for different patient cohorts. (**C**) ROC curve for HCC versus noncancerous HCC EV Z score

Sun Na et al. developed a specific extracellular vesicle (EV) purification system for hepatocellular carcinoma (HCC) based on covalent chemistry, which can quickly and effectively purify HCC EV with complete mRNA information(Fig. 7A) [98]. The author used this system to analyze the plasma samples of HCC patients and the control group. The results show that the system can accurately distinguish HCC patients from healthy people and other malignant tumor patients (Fig. 7B C). To sum up, mRNA detection based on HCC EV is expected to significantly enhance the ability of early detection of HCC in current HCC diagnosis methods.

Studies have shown that the circulating microRNA contained in the EVs content is also a potential biomarker for patients with hepatocellular carcinoma. Hyo Jung Cho et al. tested serum xenografts for expression of selected oncogene miR and evaluated their diagnostic performance [99]. The results showed that a serum combination based on EXO-MIRI-4661-5P could be used for the diagnosis of hepatocellular carcinoma.

##### Colorectal Cancer (CRC)

Colorectal cancer is a malignant tumor of the gastrointestinal tract, and the global incidence rate is on the rise. Disease detection is inefficient due to the lack of early symptoms and late metastasis, leading to higher mortality [100]. Therefore, accurate and early detection is of great significance for the prevention and control of CRC.

In recent years, RNA and protein of EVs have been widely used as new biomarkers of colorectal cancer. Through protein omics analysis, many differentially expressed proteins of EVs have been identified. Researchers found that abnormal expression of EV miRNA was a candidate molecule for the diagnosis of colon cancer patients by sequencing small RNA. Wang et al. tested this hypothesis in colon cancer patients and healthy volunteers [101]. Their results demonstrated circulating plasma EVs mRNA as a diagnostic biomarker for early colon cancer.

Similar studies have shown that specific EV-miR is also a diagnostic biomarker of CRC. Chan-Keng Yang et al. analyzed the small RNA spectrum in EVs and identified 15 characteristics of EV-miR for the detection of CRC metastasis and long-term therapeutic effect [102]. Among them, EV-miR-320c has excellent diagnostic ability and treatment monitoring performance and is a powerful clinical diagnostic marker.

#### Reproductive system diseases

Human reproduction, pregnancy and embryonic development require precise, fine-tuned and dynamic intercellular communication. Amniotic fluid, blood and breast milk all contain EVs with specific functions, so detecting these special EVs can enable researchers to detect reproductive system cancer early [3].

##### **Breast cancer**

The early detection of breast cancer (BC) has important clinical significance for cancer treatment and overall survival rate. At present, commonly used clinical breast cancer biomarkers, such as carcinoembryonic antigen (CEA), carbohydrate antigen CA125, CA153 and so on, lack sensitivity and specificity for the diagnosis of breast cancer [74]. Therefore, there is an urgent need to find new biomarkers for the early diagnosis of breast cancer.

Among all the emerging biomarkers, EVs can carry a variety of functional molecules (such as proteins, lipids, RNA and DNA fragments) inherited from their parent cells [103]. Due to its high concentration and stability in blood circulation, tumor-derived EV is considered as a promising biomarker for liquid biopsy of breast cancer patients.

Junli Zhang et al. used anti-CD9 antibody engineering biochip to capture EV in clinical plasma samples for ultra-sensitive digital detection and multiple whitening analysis [50]. The authors distinguished breast cancer patients by detecting and analyzing heterogeneous proteins of a single EV in clinical samples, which proved that EVs can be used as a biomarker for the diagnosis of breast cancer.

In order to solve the problem that EV protein markers are easily confused with soluble protein markers in peripheral blood, Tian Fei et al. developed a thermophoretic aptamer sensor (TAS) for analyzing the cancer-related protein spectrum of plasma EV [12]. At the same time, the expression levels of eight BC-related EV protein markers were analyzed using machine learning algorithm to identify the EVs characteristics, thus differentiating breast cancer patients and healthy blood donors with high accuracy.

In addition, sEV-derived mRNA in the blood of patients with breast cancer is considered to be a biomarker for the diagnosis of breast cancer. Subsequently, Liu et al. analyzed and detected mRNA derived from four extracellular vesicles (sEV) in combination with machine learning to analyze and detect mRNA derived from sEV, and the results showed that breast cancer could be diagnosed [104].

##### **Bladder cancer**

Bladder cancer is the second most common malignancy of the urinary system. Since about 80–90% of bladder cancer patients present with painless hematuria or frequent urination during the course of the disease, early diagnosis and prognosis are extremely difficult. Therefore, early detection of bladder cancer is crucial to reducing cancer-related mortality [105].

Recent studies have shown that EVs isolated from human urine can be used for cancer diagnosis and treatment monitoring [106]. Liang et al. developed a microfluidic device with integrated dual filters [107]. Bladder cancer was diagnosed by collecting and determining the concentration of EVs in urine of bladder cancer patients and normal people, and the results showed that the concentration of EVs in urine of bladder cancer patients was higher than that in other groups, indicating that EVs concentration in urine could be used for cancer diagnosis.

In addition, Juan Pablo Hinestrosa et al. measured the concentrations of related protein biomarkers in purified EV samples from patients and normal subjects. In their study, the biomarkers of the purified EV proteins successfully predicted bladder cancer [108].

In summary, EVs have become an integral part of disease and tumor mechanisms, mediating intercellular communication and participating in the occurrence and development of tumors and cancers through specific means. Through continuous exploration, decoding and detection of EVs contents, EVs gradually become a new diagnostic biomarker.

### Central nervous system diseases

The normal function of the central nervous system depends largely on the exchange and integration of information between neurons, microglia, astrocytes, and oligodendrocytes. The communication between nerve cells affects the development of the brain and the stability of the internal environment. It is also related to the occurrence of nervous system diseases such as neurodegenerative diseases. Nerve cell-derived EVs are an important signal carrier in the central nervous system. EVs play an important role in intercellular communication and neurodegenerative disorders by carrying bioactive molecules and mediating the signal transduction between adjacent or distant cells. In addition, neuronal EVs can also cross the blood-brain barrier, indicating that EVs can serve as a potential window for understanding the physiological and pathological activities of the brain [109].

#### Alzheimer’s disease

Alzheimer’s disease (AD) is a neurodegenerative disease with a hidden onset and progressive development, which mainly occurs in the elderly population, and its prevalence increases significantly with age. The main manifestations of patients are cognitive or behavioral (neuropsychiatric) changes such as memory. At present, AD diagnosis and disease detection are subjective and late, and they are mainly realized through clinical and neuropsychological evaluation [110].

Recent studies have suggested that EVs may play an important role in the pathogenesis and progression of AD. Plasma EVs is associated with pathological AD proteins and is abundant in amyloid plaques of the human brain [111]. Therefore, capturing this exchange of EVs binding information could provide a blood-based conversion opportunity for the molecular characterization of AD.

**Fig. 8:**
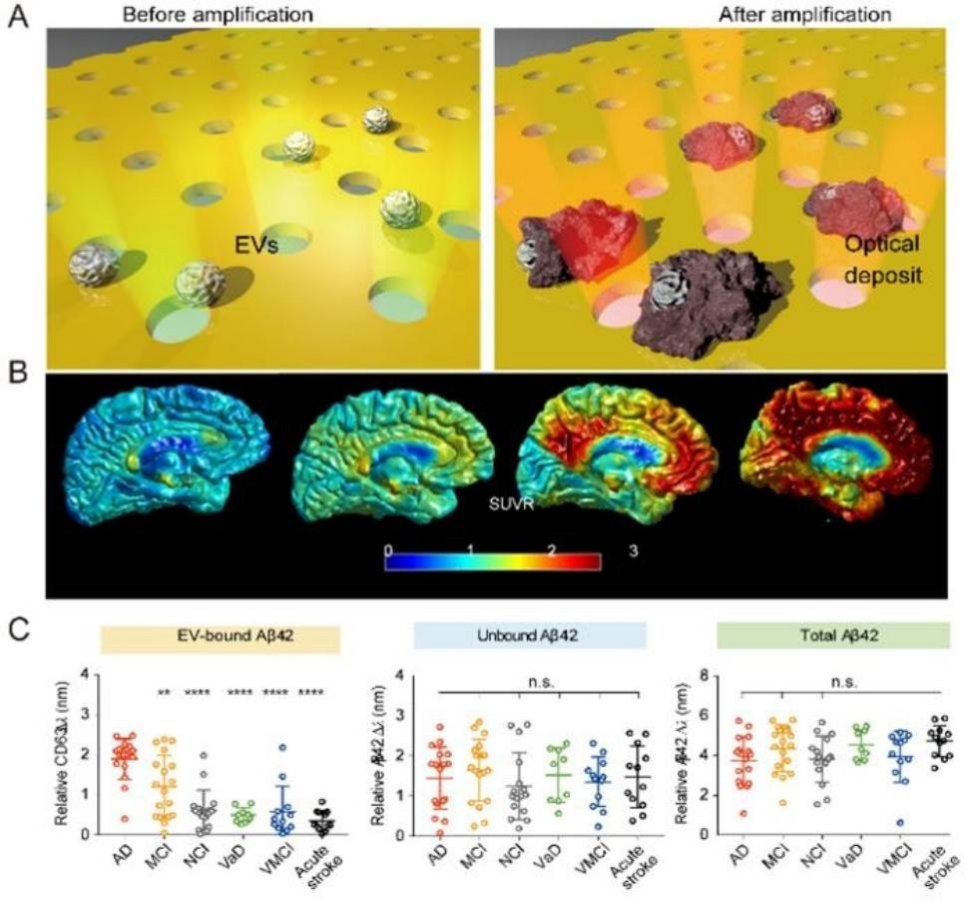
APEX platform for analyzing circulating EV-bound Aβ and reflecting association with cerebral plaque burden. (**A**) APEX platform analysis and detection principle. (**B**) Clinical correlation between circulating EV binding Aβ and brain imaging. (**C**) Analysis of Aβ42 population with different circulation in different clinical groups

Carine Z. J. Lim et al. developed a highly sensitive analytical platform (APEX) for multi-parameter analysis of EVs-bound Aβ directly from plasma(Fig. 8A) [112]. Compared to routine testing, APEX was able to directly determine circulating Aβ42 in blood samples (Fig. 8B). And distinguish different clinical populations (Fig. 8c). Their results showed that EVs combined with Aβ population strongly reflected the deposition of cerebral plaques.

In addition, Maria Serpente et al. isolated small neurogenic EV (NDEV) from plasma of AD patients and healthy subjects and further analyzed their miRNA contents [113]. The detection results showed that the miRNA of the related specific EVs varied with the changes of the detected subjects, indicating that it could be used as a diagnostic biomarker for Alzheimer’s disease.

#### Glioblastoma Multiforme (GBM)

Reproduced with permission from Lim, C.Z.J et al [112]. Copyright Nature Publishing Group(2019).

Glioblastoma is a highly aggressive and angiogenic tumor that produces high levels of proteins such as VEGF, FGF and PDGF, which can induce the proliferation of endothelial cells, where cancer cells can be converted into endothelial cells or pericytes, which ultimately break the blood-brain barrier (BBB) and cause treatment failure. Therefore, timely diagnosis and accurate treatment of gliomas are very important [114]. Studies have shown that GBM releases a large amount of tumor-specific EVs into the systemic circulation, including mRNA, miRNA and other contents.

**Fig. 9:**
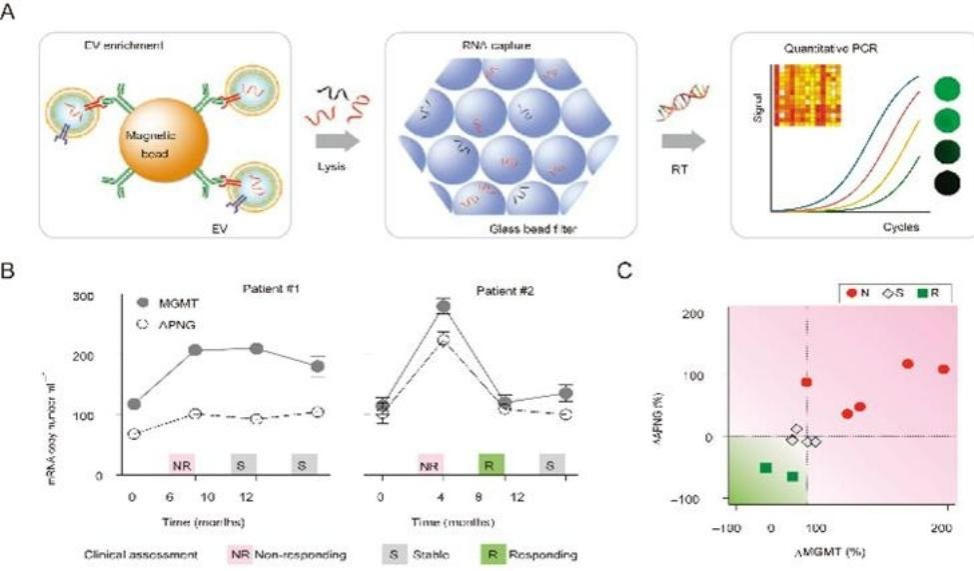
Component of microfluidic platform and analysis of clinical samples. (**A**) Microfluidic platform structure. (**B**) Changes in mRNA levels in longitudinal EV associated with clinical assessments. (**C**) Changes in EVs mRNA levels in GMB patients after TMZ treatment

Reproduced with permission from Huilin Shao et al [115]. Copyright Nature Publishing Group(2015).

Huilin Shao et al. developed a sensitive microfluidic platform for the enrichment and rapid analysis of RNA content of cancer-specific EVs in blood (Fig. 9A) [115]. Analysis of EVs blood MGMT and APNG mRNA using this platform in multiple GMB patients revealed that mRNA levels varied from patient to patient, significantly correlated with the final treatment outcome (Fig. 9B), and predicted treatment outcomes (Fig. 9C). Their results suggest that sensitive and thorough detection of EVs-associated mRNA markers can provide a timely diagnosis and prognosis for patients with GBM.

To explore whether protein in EVs can be used as a potential clinical diagnostic marker of GBM, Yang et al. tested the protein secreted by EV in the blood and cerebrospinal fluid of the xenografts in situ mouse model and found that DNM3, p65, and p53 could be detected, which was related to disease progression and survival rate, indicating that EVs can be used as a potential clinical diagnostic marker of GBM [116].

As mentioned above, EVs have been shown to be a participant in central nervous system disorders and a valuable clinical diagnostic biomarker. As EVs can cross the blood-brain barrier as a carrier of communication between the central nervous system and the periphery, EVs stand out from other detection and treatment modalities in the assessment and treatment of nervous system-related conditions. However, the role of EVs in the central nervous system is still in the initial stage, which still needs to be continuously explored and analyzed.

## Conclusion and future perspectives

Research on extracellular vesicles (EVs) has developed rapidly over the past decades, but many challenges remain in the extraction and analysis of EVs. Separation and enrichment of EVs for biomedical research and clinical applications is crucial [117]. In this review, we introduce various new methods for isolation, purification, and enrichment of EVs that enable faster and more convenient recovery of highly purified, large amounts of EVs faster and more conveniently [14, 30].

Additionally, we describe various new detection technologies developed in recent years, including thermoelectric biosensors, nano plasmonic biosensors, droplet PCR, membrane fusion, and single EV detection. These methods effectively solve the problem of difficult detection and analysis of information in EVs and can help researchers better understand the information provided by EVs through proteins and nucleic acids. We also briefly describe the diagnostic applications of EVs in different diseases in various human systems, demonstrating the excellent performance and good prospects of EVs as diagnostic biomarkers [118, 119].

### Single EV detection

Even with complex separation and enrichment methods, the required target EVs may need to be more abundant for complete detection and analysis. However, by detecting the expression of proteins or mRNA at the level of single EV, we can effectively understand the heterogeneity of EV structure and composition, obtain more information from a limited number of EVs, and promote its application in medical diagnosis and treatment [75, 120]. The detection of biomarkers at the level of individual EVs will significantly improve the prognosis and diagnosis of EVs and enhance our understanding of their phenotype, biogenesis, and function. For cancer patients, the earlier the disease is detected, the greater the chance of survival. For example, for breast cancer, the overall 5-year survival rate of women with stage I breast cancer is 99%, while that of women with stage IV is 29%. The advantage of single EV analysis is that it can solve many cancers that cannot undergo early and effective screening. In breast cancer, only 61% of cases were diagnosed at early stages (stage I and II). Undoubtedly, the use of single EV analysis to diagnose early clinical cancer can save countless patient lives.

### Integrated system

EVs have the advantage of non-invasive detection of various cancers and brain diseases in the field of detection. Still, nowadays most existing methods are complex, time-consuming and require expensive equipment and trained professionals [121]. One of the ways to improve in the future is to combine multiple steps into one platform and perform them automatically to reduce the time and material costs of EV detection [122]. The integrated platform with high automation and rapid processing will effectively promote the application of EVs in clinical field.

In summary, the development of EV enrichment technologies and the latest vesicular protein and nucleic acid biological information decoding technologies provide powerful tools for studying the role of EVs in biological processes and their application in clinical diagnosis. As a potential biomarker, EVs have demonstrated great potential and broad prospects in the diagnosis of various diseases.

## Data Availability

It is not applicable to this article as no new data were created or analysed in this study.
